# An Internet of Medical Things Cyber Security Assessment Model (IoMT-CySAM)

**DOI:** 10.7759/cureus.94639

**Published:** 2025-10-15

**Authors:** Faouzi Jaidi, Sondes Ksibi, Adel Bouhoula

**Affiliations:** 1 Innov’COM\Digital Security Research Lab, Higher School of Communications of Tunis, University of Carthage, Tunis, TUN; 2 National School of Engineers of Carthage, University of Carthage, Tunis, TUN; 3 Department of Next-Generation Computing, College of Graduate Studies, Arabian Gulf University, Manama, BHR

**Keywords:** cyber security, healthcare security, iomt security, privacy and risk assessment, risk management

## Abstract

Backgrounds

E-health systems and particularly those based on connected medical devices, commonly known as Internet of Medical Things (IoMT) paradigm, are among the trends these days. As a transformative technology in the healthcare domain, it mainly enables real-time monitoring and seamless data exchange. However, given their complexity of architecture, their ubiquitous nature, and their resource limitations, ensuring the privacy and protecting user’s data in such systems remains issue of concern. New security risks within Internet of Things (IoT)/IoMT-based e-health applications have emerged.

Methods

The current research work relies on an in-depth study of IoMT architectures addressing their technical foundations as well as their associated security considerations. From a security standpoint, common vulnerabilities, threats, and associated risks are identified, and state-of-the-art mitigation strategies, mainly standard risk management frameworks, are evaluated. A comparative analysis was conducted on existing solutions, discussing their suitability in addressing the identified concerns.

Results

The conducted study reveals that, in general contexts, applying and conducting risk management processes are not easy tasks (seem to be confusing and prone to errors), especially within heterogeneous and complex systems such as IoMT applications. There is a strong need for automatic solutions to simplify the complexity of the application of different models and processes. Automation and tools are considered among important factors to ensure the success of any proposal. We present, in this manuscript, our framework designed to handle this issue and introduce our system called IoMT-CySAM (Internet of Medical Things - Cyber Security Assessment Model), a main part of our research work in this context. IoMT-CySAM allows evaluating trustworthiness and managing cyber risks within IoMT environments.

Conclusions

As the deployment of IoMT systems faces critical security and privacy challenges, automated risk management solutions are highly required to handle the issues. The IoMT-CySAM, as a context-aware and adaptive solution, is defined to help with trustworthiness evaluation and security risk management within IoMT environments. Moving forward, research should prioritize automated reliable solutions that ensure both effective protection and operational efficiency.

## Introduction

While traditional healthcare is inflexible and time and cost demanding, Internet of Things (IoT) is expected to revolutionize patient care pathways by providing remote and precision healthcare. IoT application in healthcare, commonly known as e-health or digital health, is a distributed infrastructure that relies on sensing devices (blood glucose monitor, temperature and motion sensors, etc.) and wireless communication technologies (Wi-Fi, RFID, Bluetooth, etc.) for more precise diagnosis, treatments, and faster handling time.

While e-health refers to a broad set of services and technologies, Internet of Medical Things (IoMT), as a subset of IoT, refers to connected devices and applications that enable them to function [1]. IoMT utilizes a variety of sensors with software and connection capabilities for the implementation of connected healthcare systems. As a widely decentralized and intelligent infrastructure, IoMT facilitates the integration of various medical devices, services, and applications for efficient medical data exchange [2]. By offering continuous monitoring of vital signs, increasing mobility, improving flexibility, and saving time, this technology enhances healthcare delivery. From basic fitness tracking to advanced surgical procedures, IoMT delivers more precise and timely outcomes [1]. As a transformative force in healthcare, IoMT holds immense potential for healthcare innovation and advances the sector's digital revolution [3].

Although it offers considerable benefits, the IoMT paradigm also introduces crucial concerns mainly regarding data security and patient privacy [4]. The system’s interoperability, compliance to standards, openness, and complexity arise as challenging issues to safeguard privacy and security. Medical data are sensitive and highly regulated; therefore, healthcare providers are responsible for preventing it from being compromised. However, IoMT devices bring a certain level of risk due to the considerable amount of sensitive data being exchanged between patients and healthcare professionals. The loss of confidential medical data could be embarrassing or damaging. Cybercriminals can target specific medical devices (e.g., smart pacemaker, glucometer, or insulin pump) to obtain medical information, disable them, or make them as an entry point to compromise other systems. Security risks in IoMT are supposed to be of the most significant barriers to the expansion of e-health. Hence, securing IoMT is of paramount importance for healthcare organizations and industrial companies. An increasing interest from researchers in developing new security mechanisms or strengthening existing approaches is growing and being noticeable in recent years [4-5].

Risk management methods, as parts of enterprises management processes, aim to establish an effective control of the incurred risks. Those approaches have been proposed in the context of IoMT security assessment. Their main limitations are the subjective reasoning of descriptive models (of qualitative evaluation of risks) and the high complexity of numerical models (of quantitative evaluation models). Moreover, as risk management is extensive and demanding, automating tools fail to cover all the process steps, making a comprehensive and thorough risk analysis a very hard task.

In general contexts, applying and conducting risk management processes are not easy tasks, especially within heterogeneous and complex systems such as IoMT applications. These tasks seem to be confusing and prone to errors [2]. The need for automatic solutions and tools is highly required to simplify the complexity of the application of different models and processes. Automation and tools are considered among important factors to ensure the success of any proposed framework.

In the current paper, as a primary goal, we especially deal with the presentation of our solution for enhancing trustworthiness and help with making decisions within e-health environments. A concrete result derived from our approach is the definition of the IoMT-CySAM, ensuring an efficient, reliable, and easy-to-use cyber-risk management framework within IoMT infrastructures. As a secondary goal, we refer to a case of application to support the evaluation of the proposed model’s efficiency and applicability.

In the reminder of this article, the main IT advances and their application in the medical domain are presented and discussed, the background of this research work and its related works are outlined, and the presentation of our IoMT-CySAM and its application in the security assessment of IoMT infrastructures are detailed. Finally, the summary of this research work and its perspectives are highlighted.

## Materials and methods

IT advances and their application in the medical domain

Recent IT advances in many technological fields contributed to the development and spread of medical and healthcare applications. In the following, we cast a glance on some main advances.

Pervasive computing boosts the development of medical and healthcare solutions. Unlike desktop computing, a trend to integrate computational and communication capabilities into everyday objects has driven the growth of pervasive or ubiquitous computing. These devices combine the physical and virtual worlds and contribute to the development of the IoMT paradigm.

With the ubiquity of connected devices and the diversity of applications, large amounts of data are generated and exchanged. Some applications require real-time data exchange. Connected devices, which are initially limited in processing and storage resources, are unable to handle the volume of collected information. This need is the basis of the concept of cloud computing. As a new concept in computing that promotes the development of IoMT applications, the cloud computing allows on-demand access to resources such as storage, processing, and computing through configurations in terms of infrastructures, services and/or applications [6].

The growth of computing and storage capabilities of connected objects led to an optimized method to process data at the network edge called edge computing. It no longer requires outsourcing processing and storage to the cloud in order to minimize bandwidth requirements and undertake analysis as close as possible to data sources. Edge computing decreases the exposure of data to different cloud threats.

Wireless sensor networks (WSNs) are gaining increasing attention worldwide as they are a technological key of IoT. Platforms integrating processing, storage, sensors, and wireless networking have recently emerged to offer the ability to sense a physical phenomenon in difficult-to-access locations. Wireless communications are convenient in such situations. Clusters of smart sensors operating in networks with minimal or no infrastructure and are highly constrained in terms of resources (i.e., power, radio range, processing). WSNs have various applications in healthcare; wireless body area networks (WBANs) emerged as a cost-effective and promising solutions for monitoring human physiological sign. Sensors, placed around or inside the body, have numerous uses such as fitness monitoring, rehabilitation, assisted living, and remote health monitoring. Healthcare organizations use WBANs to track patients' biological indicators such as blood pressure, temperature, blood oxygen saturation, glucose level, and heart rate. These physiological data of the patient that come from the sensors are transmitted via a smart phone or tablet to a base station in the radio network and then sent to a solution dedicated to processing these data in a remote site. The medical staff is then be able to make use of this data. WBANs are a basic part of technological boosters of healthcare services.

Internet of things in healthcare: the IoMT context

The IoT covers several smart domains (smart homes, smart cities, smart transportation, smart health services, etc.). In the rest of this paper, we are specifically interested in the health sector. In the medical field, connected digital devices improve every step of the patient care pathway [7-8]. The centralization of data in the cloud, the continuous updating of medical information, and the management of access to data are vectors of economic gain [9].

Wearable devices can collect data about a person's state of health and transmit it, continuously or over specific periods of time, to healthcare professionals. For example, a smart watch with built-in blood pressure or heart rate sensors can be used to detect or prevent cardiac arrhythmia or stroke symptoms [10]. Real-time monitoring of diabetes is also made possible using connected devices [11]. For elderly people, connected medical objects bring added value in relation to the monitoring of their well-being and comfort and emergency states such as falling.

During periods of health crises, like in the COVID-19 pandemic, connected medical devices have proven to be effective, as they make it possible to ensure continuous monitoring of patients while avoiding overcrowding in hospitals. Historically, medical care was centralized and mostly delivered in hospitals. Recently, and thanks to connected medical devices, which have become increasingly intelligent and ubiquitous, the healthcare sector has undergone a shift in the manner services are delivered from systems that rely solely on face-to-face human diagnosis to solutions that rely solely on technology [12]. As a result, innovative solutions for the follow-up and treatment of patients at home have emerged. They are based on devices that collect information about people's health status and transmit it via the network to caregivers or other relevant persons (such as a family member). The information collected is stored to be analyzed by specialists in order to make diagnoses and decide on appropriate actions. Patients themselves can view the collected indicators in real time. Some chronic diseases require continuous monitoring of vital signs in order to prevent incidents and allow medical staff to intervene in time and avoid complications. The storage of medical data, most often in the cloud, makes it possible to establish a large database, advantageous for pathology studies and research [12]. Connected medical devices offer a solution that meets the medical assistance needs of elderly people and people with reduced mobility who want to stay at home. Healthcare has therefore undergone a radical transformation through the IoT, and we are talking more about e-health [13].

The applications of IoMT, also known as IoT for e-health or, in some instances, medical Internet of things (MIoT), establish a heterogeneous ecosystem consisting of various connected medical sensors and devices, clinical systems, and specific medical software. The goal is to improve the quality of medical treatment with a reduction in effort, time, and costs.

IoMT applications enable the integration of smart technologies into medical devices for more effective disease monitoring and patient follow-up. Over the past two decades, connected medical devices have continued to revolutionize the way healthcare is delivered. Connected medical devices include implantable devices, wearables, diagnostic devices, therapy devices, health applications, and objects used in the event of hospitalization. Personalized, real-time care is offered to patients, accelerating the expansion of the IoMT paradigm. Indeed, the recent Fortune Business Insights report published in 2023 forecasted the IoMT market to grow by 26.1% between 2023 and 2030 [14].

Smart medical devices minimize the risks of human error and provide a more accurate care service while ensuring the reduction of healthcare costs and time and patient engagement in the healthcare delivery cycle. This has become possible via connected devices that collect medical data that is accessed directly by the patient. One of the clear improvements of these applications is the early detection of health complications. For example, patients with cardiovascular diseases can be monitored in real time through a connected heart rate monitor without the need to hospitalize them [12].

Connected medical objects also make it possible to improve patient engagement in the care cycle they receive through the possibility of consulting their medical information at any time and interacting with healthcare staff more easily. For example, health applications can provide reminders about the doses and times of medication, alert the patient or a family member if there is a complication of their health condition, and provide therapeutic follow-up to the patient and adjust the patient's care plan if necessary.

Cyber-risk management for critical systems

While networked systems are being more complex and attacks against them are developing rapidly, security controls are unable to cope with their ever-changing threats. High level structured security assessment methodologies such as risk management methodologies are proposed to better deal with this issue. We discuss, in this section, the need for risk management and the main activities to apply it on critical systems.

The Need for Security Risk Management

The complexity of networked systems, the over growing number of attacks against them, and the high cost of available security solutions have emphasized high-level structured solutions to evaluate the security of such systems and identify the most convenient defense solutions. In recent years, many critical activities are dependent on information technologies, and organizations rely on the Internet and their networks for various business activities. The impact of a security breach can have ruinous consequences on the organization. Therefore, managing risks associated with information technology became a top management issue [2].

Risk management is an old concept applied to computer security assessment many decades ago. Various guidelines, standards, and best practices rules for security risk assessment have been published by standardization organizations and other relevant organizations.

Security risks are increasingly becoming more challenging, but risk management frameworks preserve the foundation of basic components, including risk identification, analysis, and management plan, to mitigate risks to an acceptable level.

Overview of Concepts

Modern enterprises are increasingly relying on automated and distributed information systems to be competitive and expand their business far from borders. However, they are facing serious challenges to preserve the security needs of their networked systems. These needs should be regarded according to the evolving complexity of security analysis. Fast and effective decision-making is therefore of high importance. Risk management methods support decision-making in such complexities and ever-changing environments. It can ensure support for both security professionals and managers and provide them with a tool to figure out the system security state and recognize risks, to identify defense solutions according to risk evaluation and prioritization in order to select the most convenient security mechanism in terms of efficiency and cost, and to define response plans to mitigate risks rapidly and effectively when an event occurs.

Despite having all these benefits, risk management is still a hard task and require a certain level of abilities and involves several steps such as asset analysis (identifying assets and prioritizing them), threat and vulnerability identification, impact evaluation of security events, and defense control selection.

Generic Risk Model

**Figure 1:**
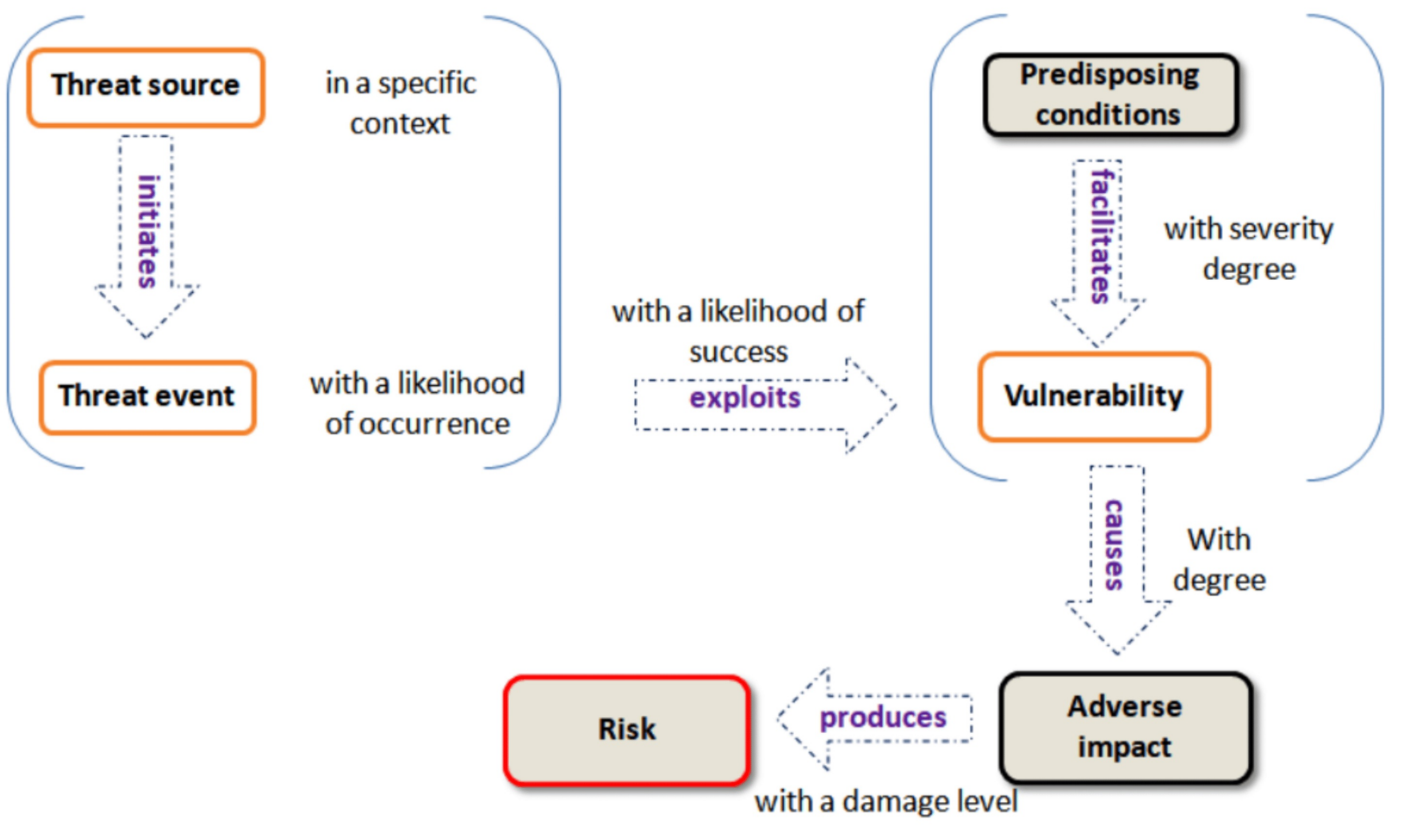
Generic risk model The risk management model is conceived as a holistic process that encompasses basic steps: risk identification, risk analysis, and risk evaluation. This process integrates fundamental sub-processes such as threat analysis, vulnerability identification, and impact assessment.

Security assessment is basically done according to standardized management practices following a risk-based approach that consists of managing potential security risks (i.e. effects of a potential event) on a given system. Security risk analysis is a set of activities undertaken to manage potential sources of threats against a system to reduce its exposure (vulnerabilities) to breaches and mitigate the relative consequences (impact) of successful events. Well-known standardization organizations such as ISO [15] and NIST [16] defined risk management as a holistic process of security risk identification, analysis, and evaluation through a combination of sub-processes such as threat analysis, vulnerabilities identification, and impact assessment (Figure 1).

Review and analysis of security assessment models

Security Assessment of IoMT

In the IoMT context, the ISO-14971 [17] defines the procedure of medical device risk identification for manufacturers. The main objective of this publication is to help in evaluating risks, applying mitigation measures, and monitoring the effectiveness of the applied procedures. This standard introduces severity values as qualitative metrics for impact assessment. The ISO-24971 publication [18] gives guidance in applying the ISO-14971. The International Electrotechnical Commission IEC-80001 [19] is a complementary relevant publication helping medical devices manufacturers and health organizations to apply risk management for medical device networks. The NIST published SP 800-30 [20], which is a special publication offering mainly guidance on how to carry out risk assessment and develop mitigation measures. The special publication NIST SP1800-8 [21] focuses on security of wireless infusion pumps and extends the general-purpose risk management standards and guidelines. The publication presents a security in-depth methodology to protect the pump, the server, and the network. It provides a list of common threats and vulnerabilities against such medical devices. The NISTIR 8228 Report [22] highlights the differences between IoT devices and conventional IT when assessing risk. The OWASP [23] provides a checklist, for medical devices, displaying typical vulnerabilities that need to be verified. FAIR [24] is defined as a quantitative framework to help organizations in analyzing information security and operational risks in financial terms. MITRE is widely recognized for developing cybersecurity frameworks, particularly the MITRE ATT&CK [25], a knowledge base of adversary techniques used for threat intelligence, security assessments, and incident response.

As illustrated in Table 1, the majority of the publications consist of guidelines and best practices focusing primarily on managing risks that arise after system deployment (post-deployment risks). Their main objective is to provide guidance about how risk assessment could be conducted and how qualitative evaluations (i.e., minor, low, medium, and high) could be used to assess the impact of cyber threats on medical devices.

**Table 1:** Summary of risk assessment and management standards ISO, International Organization for Standardization; IEC, International Electrotechnical Commission; NIST, National Institute of Standards and Technology; OWASP, Open Worldwide Application Security Project; FAIR, Factor Analysis of Information Risk; MITRE ATT&CK, MITRE Adversarial Tactics, Techniques, and Common Knowledge

Organization	Publication	Type	Scope	Approach	Application
ISO	ISO-14971 [17], ISO-24971 [18]	Standard	Risk analysis, risk management	Qualitative, quantitative	Pre-deployment, post-deployment
IEC	IEC-80001 [19]	Standard	Risk analysis, risk management	Qualitative	Pre-deployment, post-deployment
NIST	NIST SP1800-8 [21], NISTIR 8228 [22], NIST SP 800-30 [20]	Standard	Risk analysis, risk management	Guidelines	Pre-deployment, post-deployment
OWASP	OWASP [23]	Standard	Risk management	Qualitative	Post-deployment
FAIR	FAIR [24]	Framework	Risk analysis	Quantitative	Pre-deployment, post-deployment
MITRE	MITRE ATT&CK [25]	Guide	Risk analysis	Qualitative	Pre-deployment

Classification and Comparison

Several frameworks, driven by different motivations, have been developed for security risk assessment purposes. In terms of evaluation techniques, there is no clear boundary, as both qualitative and quantitative evaluations are commonly used to assess risk.

Generally, the risk management methodologies can be divided into two main phases: risk assessment and risk mitigation. Risk assessment covers the sub-processes of threats, vulnerabilities, and impact analysis, while risk management involves the sub-processes of prioritization, mitigation, and effectiveness evaluation. Regarding the development life cycle of a medical device, risk analysis may be applied to two main phases: pre-deployment (before utilization) and post-deployment (when operating). Some standards may cover the complete range of IoT devices, while others are IoMT-specific or may only be dealing with a particular type of medical devices.

IoMT-CySAM

Goals

Since e-health environments are dynamic, ubiquitous, highly constrained, and complex by nature, they are under various and highly changing threat models. A variety of risk management contexts may fit to specific situations or may not fit (at all or with an appropriate manner) to diverse other cases. A reliable solution to enhance the security and preserve the privacy within IoMT-based e-health applications has to effectively deal with associated emerging cyber risks. To ensure a high level of efficiency, we propose a context-aware solution that mainly aims to ensure, for a global e-health service delivery process, an effective evaluation of the cumulative risk (end-to-end risk); establish, based on a set of context-specific risk metrics, qualifiers, thresholds, factors, etc., a fine-grained risk management process; and automate the update procedure for the risk mitigation response.

Principle and Approach

To meet the previously set objectives, our solution relies on a fine-grained approach that segments the entire risk zone to three distinct areas: (1) data acquisition area (devices), (2) information gathering and transmission area (communications), and, finally (3) data processing and storage area (typically databases).

**Figure 2:**
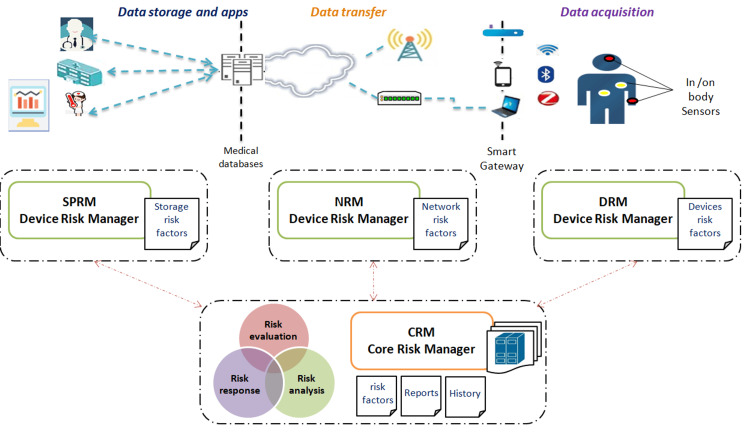
Risk management approach Four foundational agents (subsystems) form the basis of our risk management approach: the device risk manager deals with risk management in the data acquisition layer, the network risk manager is responsible for managing risks in the network layer, the storage and processing risk manager performs the risk management for the data storage and processing layer, and the core risk manager, as an orchestrator, performs the end-to-end risk management. CRM, Core Risk Manager; DRM, Device Risk Manager; NRM, Network Risk Manager; SPRM, Storage and Processing Risk Manager

The principle of our approach (Figure 2) relies on the following agents or subsystems: a Device Risk Manager (DRM), a Network Risk Manager (NRM), a Storage and Processing Risk Manager (SPRM), and a Core Risk Manager (CRM) as an orchestrator [2]. The DRM deals with risk management for the devices in the data acquisition layer. The NRM is responsible for managing risks in the network layer of the infrastructure. The SPRM performs the risk management for the data storage and processing layer composed mainly of cloud databases. Finally, the CRM performs an end-to-end risk management. Furthermore, risk classification, prioritization, and results correlations are performed to refine the decision making process.

Identification of Anomalies

Device vulnerabilities, system abnormalities, communication breaches, and data threats have to be detected to help with setting up a proactive security by evaluating the impact of threatening scenarios on the system and prioritizing IoMT assets when applying security policies. To do so, we refer to different machine learning (ML)-based processes, allowing a real-time intrusion/anomaly detection while the system is functioning. We mainly applied diverse ML algorithms, such as the decision tree (DT), gradient boosting (GB), perceptron (P), stochastic gradient descent (SGD), linear support vector classification (LSVC), and random forest (RF), on the publicly available BoTNeT-IoT dataset gathered from a realistic IoT network traffic that contains 72 million records (describing benign and various DoS/DDoS attacks) and 23 features.

It is important to note that the used dataset cannot be directly manipulated in its unsuitable raw format since it encloses a lot of noise. Moreover, in real-world network environments, the different attack types occurrence is naturally unbalanced, while training the selected models on the unbalanced data may lead to biased results. In such cases, the models often perform well in detecting majority classes but fail to detect rare/minority scenarios, and this may lead to models overfitting/underfitting. In order to avoid overfitting/underfitting cases and ensure a reliable detection, we mainly worked to balance the data before proceeding to the next phase. After evaluating different strategies (mainly under-sampling, over-sampling, and hybrid methods) taking into consideration the specific distribution of the data and the sensitivity of our algorithms, we opted for the Synthetic Minority Over-sampling Technique (SMOTE) as a balancing technique. Moreover, we applied the LabelEncoder and One-Hot Encoding techniques for data transformation and encoding as well as the StandardScaler technique for data normalization.

Risk Assessment and Treatment

**Figure 3:**
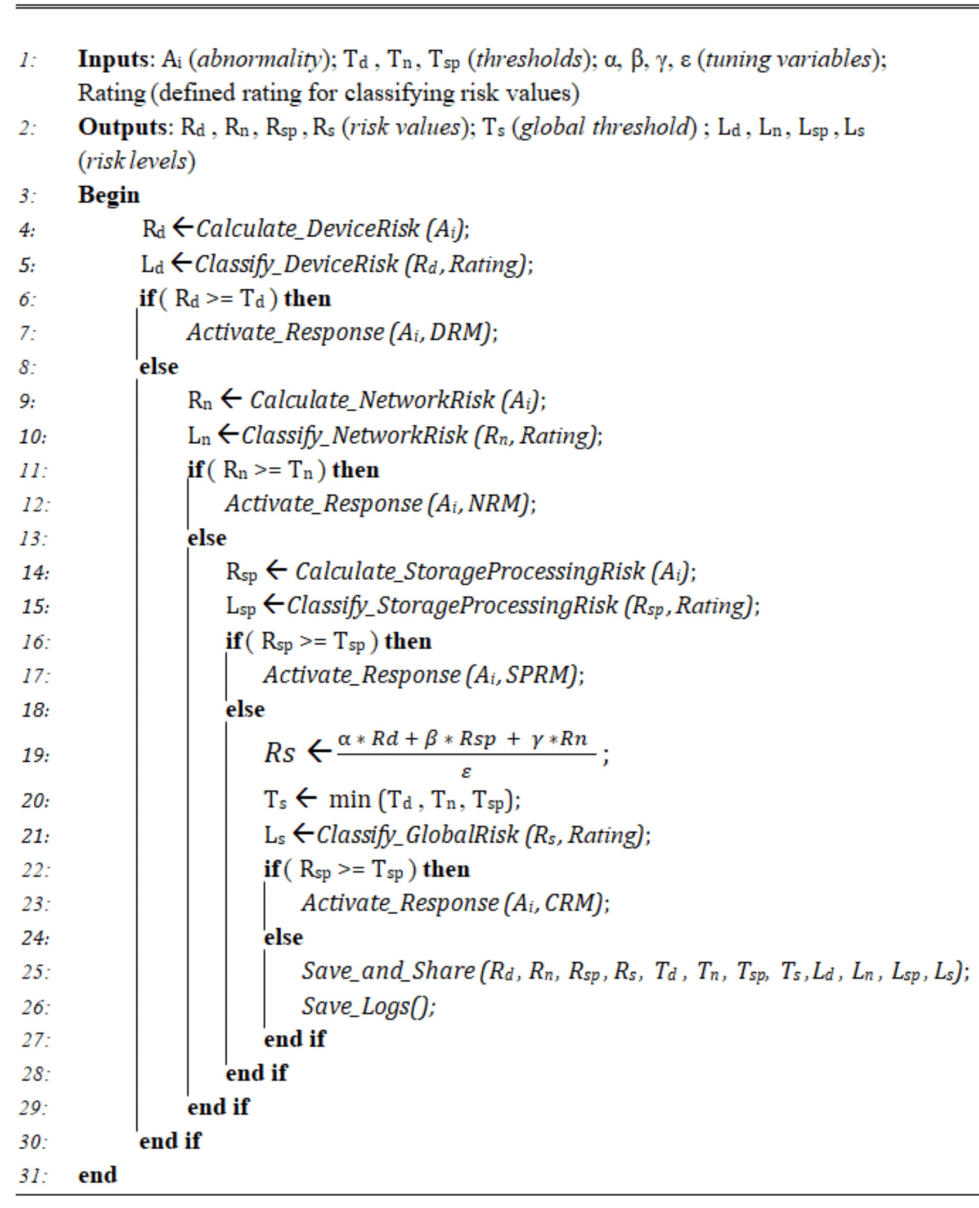
Risk treatment algorithm

To carry out an end-to-end risk evaluation and treatment within IoMT environments, our system relies on the algorithm depicted in Figure 3. The risk assessment process evaluates and classifies abnormality risks and activates associated response plans.

As for the risk classification, we consider an initial risk rating as follows: minor (≥0% and <20%); low (≥20% and <40%); moderate (≥40% and <60%); high (≥60% and <80%); extremely high "critical" (≥80%). Obtained risk values are then classified according to this rating.

The risk of an abnormality Ai (e.g., sensor failure) is evaluated according to formula 1, where P(k) represents the likelihood that the abnormal use pattern k (k= {1, . . . , m}) takes place, C(k) describes the incurred impact or cost, and CM corresponds to the value of implemented countermeasures.

 $R(A_i)=\sum_{(k=1)}^m P(k) * C(k) - CM$ (1)

Abnormalities costs are defined on a scale varying from 1 to 10 where the parameter weight 10 (is the highest scale) that belongs to the worst-case.

We determine the likelihood of development of an abnormal use to an attack based on corresponding parameters in each considered area [26].

Device area: We refer to formula 2 to evaluate the likelihood of abnormalities in this area, taking into account four parameters. S represents the readiness of the medical device to recognize (identify/detect) and address (react/respond to) the attack or abnormality Ai. K indicates the knowledge degree about the abnormality. It highlights the user's lack of security knowledge and/or trainings about the abnormality/attack Ai. D highlights the attack/abnormality difficulty level, and C gives indication about the device criticality.

$P(A_i)= \frac{S(A_i) + K(A_i) + D(A_i) + C(A_i)}{4}$ (2)

Network area: In the network area, we rely on formula 3 to evaluate the likelihood of abnormalities in this area taking into account four parameters: S indicates the security level of the communication protocols, Rs represents the resource availability of the networking nodes, N stands for the openness degree to Internet, and D corresponds to the difficulty level of attacks.

$P(A_i)= \frac{S(A_i) + Rs(A_i) + N(A_i) + D(A_i)}{4}$ (3)

Storage and processing area: We consider formula 4 to evaluate the likelihood of abnormalities at the storage and processing area taking into account three parameters: S indicates the supported and/or deployed security functions, E evaluates third parties involved in managing the stored data, and U represents the users engaged with the system.

$P(A_i)= \frac{S(A_i) + U(A_i) + E(A_i) }{3}$ (4)

The CRM, according to formula 5, evaluates the whole risk of the system with: Rs is the system global risk; Rd is the risk evaluated in the device area; Rn corresponds to the risk computed in the network area; Rsp corresponds to the risk evaluated in the storage and processing area; $\alpha, \beta, \gamma$, and $\epsilon$ are used as tuning parameters with $(\alpha + \beta + \gamma) \leq \epsilon.$

$Rs=\frac{\alpha * Rd + \beta * Rsp + \gamma * Rn }{\epsilon}$ (5)

Finally, we refer to formula 6 for the evaluation procedure of the effective global risk Reff.

$R_{eff}= \max(Rd, Rn, Rsp)$ (6)

As highlighted by the algorithm presented in Figure 3, the system, at first, evaluates and classifies the risk of an anomaly/scenario at the device level and checks it with regard to the associated predefined thresholds in the security parameters. Then, it does the same tasks a second time at the network level and a third time at the storage and processing level. In case an obtained risk value, at any level, exceeds the threshold, the system activates a preconfigured response such as aborting the communication and generating alerts.

## Results

In the current section, we mainly focus on the practical use (step-by-step application) of the system as well as the details of its different processes.

System presentation

Our system called IoMT-CySAM allows easily putting into practice our approach for evaluating trustworthiness and managing cyber security risks within IoMT environments. The web application of this framework allows a step-by-step application of the risk management process.

Specification of Risk Scenarios

To understand the reliability level of an IoMT-based e-health solution, our system offers the possibility to simulate the impact of possible abnormalities. It allows defining a set of attacks, intrusions, and/or anomaly scenarios. This serves later for evaluating, analyzing, and managing their associated risks.

**Figure 4:**
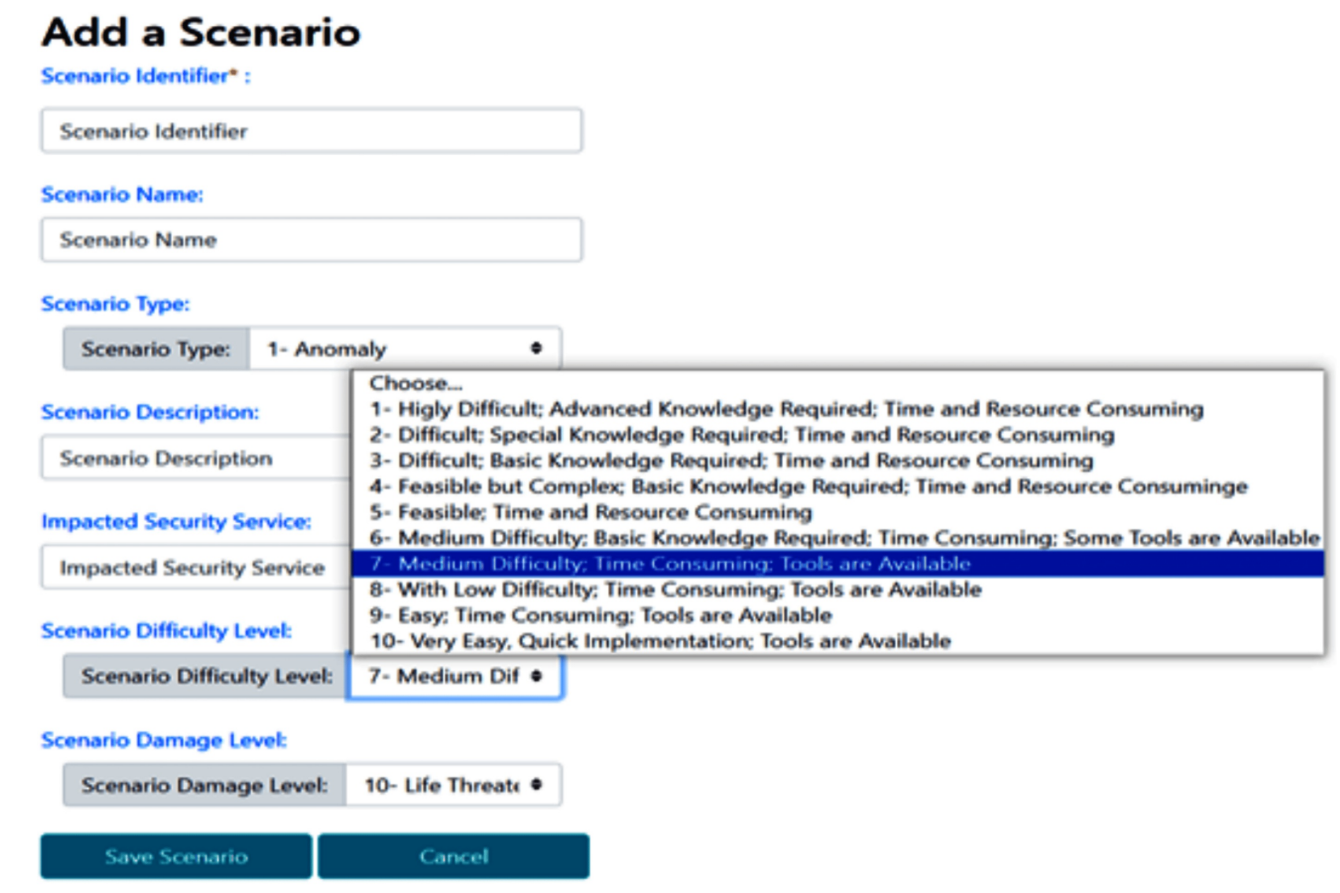
Specification of Risk scenarios This IoMT-CySAM interface is used to specify risk scenarios for subsequent simulation. The specification of a new scenario involves specifying several relevant attributes, including its type, level of complexity, the security service potentially affected, and the associated potential damages.

As a proactive security step, the system interface (Figure 4) is used for the definition of new risk scenarios. It primarily enables the specification of the scenario type, its level of complexity, potential associated damages, and other relevant attributes.

**Figure 5:**
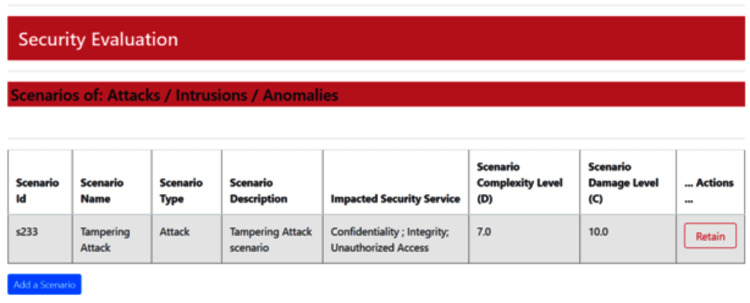
Sampless of simulated scenarios This interface of IoMT-CySAM displays the set of defined scenarios. For example, a tampering attack scenario targets the confidentiality and integrity of data handled by a medical device, potentially enabling unauthorized access to the device. This scenario is classified as moderately difficult (level 7), indicating that while it is time-consuming, the necessary tools to execute the attack are readily available. Given the potential for life-threatening consequences, the damage level has been assigned the highest severity rating of 10.

The system interface shown in Figure 5 presents the list of specified risk scenarios. It enables the updating of existing scenarios as well as the definition of new ones, based on the attributes illustrated in Figure 4.

Definition of Security Parameters

**Figure 6:**
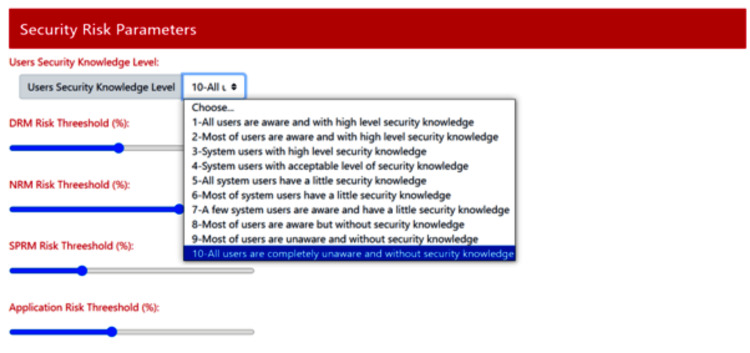
Definition of security parameters Through this IoMT-CySAM interface, the user defines required values for a set of security parameters, such as the user's level of security knowledge and the risk thresholds for the various subsystems.

In order to evaluate the impact of identified/simulated incident scenarios on the proper functioning and the security of an IoMT-based e-health solution, we need to define a set of parameters to be considered in this evaluation. As illustrated in Figure 6, this feature allows basically setting up the risk thresholds associated with each assessed area (device area, network area, and storage and processing area) to be used as decision benchmark among other security parameters.

Risk identification, assessment, and treatment

Identification of Anomalies

Device vulnerabilities, system abnormalities, communications, and data threats have to be detected to help with setting up a proactive security by evaluating the impact of threatening scenarios on the system and prioritizing IoMT assets when applying security policies.

As for operational risks, we referred to different ML-based processes allowing a real-time intrusion/anomaly detection whence the system is functioning. We mainly applied diverse ML algorithms such as the DT, GB, P, SGD, LSVC, RF, etc. on the publicly available BoTNeT-IoT dataset gathered from a realistic IoT network traffic.

In terms of experimentation, the test (after training) of the selected algorithms in the context of our BoTNeT-IoT dataset gave good experimental results and competitive performances, highlighting that even most of the implemented models achieved satisfactory outcomes, the DT and P models demonstrated superior execution time efficiency. In terms of accuracy, we noted that we obtained a 100% accuracy level with the DT, RF, and GB models [27].

Risk Assessment and Treatment

To carry out an end-to-end risk evaluation and treatment within IoMT environments, our system relies on the algorithm depicted in Figure 3. The system, at first, evaluates and classifies the risk of an anomaly/scenario at the device level and checks it with regard to the associated predefined thresholds in the security parameters. Then, it does the same tasks in a second time at the network level and in a third time at the storage and processing level. In case an obtained risk value, at any level, exceeds the threshold, the system activates a preconfigured response such as aborting the communication and generating alerts.

**Figure 7:**
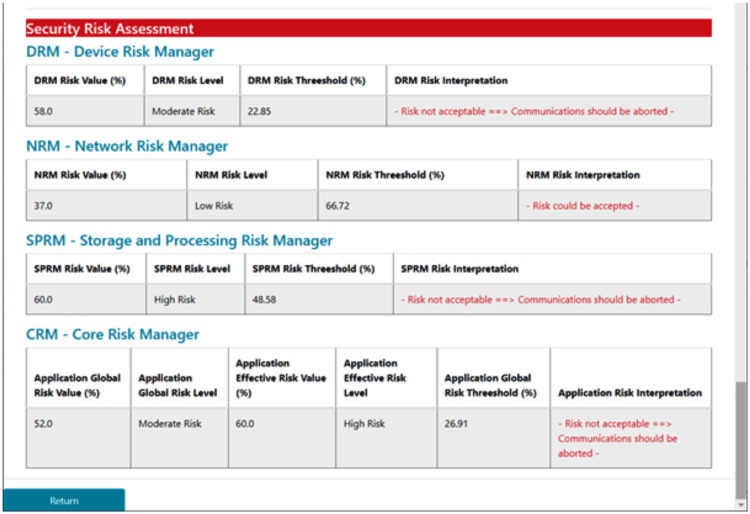
End-to-end risk treatment This IoMT-CySAM interface presents the current risk status of an IoMT infrastructure. By evaluating, classifying, and comparing the obtained risk values against predefined thresholds, each subsystem can interpret the situation and make appropriate decisions.

A global view of the situation is centralized at the core risk manager. It mainly evaluates the global and the effective end-to-end risk of the IoMT solution. Figure 7 illustrates an example of risk assessment, classification, and treatment at the different levels.

## Discussion

The integration of IoMT in medical systems has significantly advanced healthcare delivery and improved e-health solutions [2]. However, IoMT applications often operate in life-critical contexts, where even minor disruptions can have severe consequences for patient safety [7]. This introduces complex security and privacy risks that need to be carefully managed [2-4].

While traditional standards and frameworks such as ISO publications [17-18], NIST publications [20-22], and others [23-25] provide useful guidelines for organizational security and some of them (such as ISO 14971 [17]) are adopted for medical device risk analysis, they require more adaptation to meet specific IoMT constraints [28].

Our aim throughout this research work is to improve security and preserve privacy within e-health systems. This was mainly motivated by the need for mechanisms that enable the detection of emerging (new or previously unseen) anomalies and threats, the assessment and prediction of their associated potential damages, and the setup of countermeasures for a reliable mitigation [26]. There is a strong need for automatic solutions to simplify the complexity of the application of different models and processes [2].

Our solution for enhancing the security of e-health systems relying on IoMT devices defines an automated framework designed to ensure a comprehensive cyber security risk management across multiple IoMT infrastructure levels and components. It enhances existing solutions via integrating real-time monitoring, contextual risk evaluation, and dynamic decision-making. In this simulation, we introduced, technically detailed, tested, and illustrated a step-by-step application of our IoMT-CySAM with a range of IoMT devices from different smart healthcare environments. The framework demonstrated excellent performance in managing secure communication, real-time monitoring, and automated responses to anomalies or cyberattacks.

We believe that the proposed solution improves security and privacy within IoMT infrastructures in a way that minimizes costs of collateral damages that could impact the users of the system [2]. The proposal fundamentally relies on a dynamic context-aware security monitoring, an iterative risk management process, predictive analytics, a fine-grained decision-making, and a collection of up-to-date metrics. The system is flexible, thanks to its module-based architecture, and it can be easily expanded into a more fine-grained model via integrating more risk agents as necessary, especially in case of too wide or complex risk zone.

Even though it is adaptable to the IoMT context, the system’s processes require periodic updates as well as thoroughly documented description of constituent components. Depending on the available resources within the existing healthcare infrastructure, particularly in legacy environments with limited capabilities, this may represent a barrier to the system’s efficient deployment and use. From a real-world testing and deployment perspective, we are aware that, in addition to simulations, a future work is needed to particularly focus on the deployment of the system in real-world environments. This is important since it will help in (i) testing the system performance under concrete operating conditions and (ii) assessing how well the system integrates with existing solutions.

Moreover, as access to medical data is legally supervised, compliance with laws as well as legal and ethical issues should be addressed [2]. While the integration of artificial intelligence, particularly ML techniques, offers numerous benefits, it is essential to implement robust security measures to mitigate AI-specific vulnerabilities [29-30]. As explored in Houichi et al.’s study [5], safeguarding critical infrastructures requires advanced models that enhance technical solutions with policy measures, governance structures, and educational initiatives. In this way, the dynamic interplay among clinical benefits, cyber risks, security, and safety warrants further in-depth exploration [28].

By focusing on these key areas, we expect to further enhance the security, scalability, and performance of our solution, making it even more robust and adaptable to the evolving needs of modern applications.

## Conclusions

The IoMT, as a pivotal emerging technology, enables the development of distributed systems that seamlessly integrate smart medical devices, healthcare services, and software applications to enhance the exchange and management of medical data. Given that health information is highly sensitive by nature and that healthcare services play a critical role, addressing cybersecurity concerns, as well as proactive and operational risks, must be a top priority throughout the system lifecycle.

Improving security and ensuring trustworthiness of IoMT environments enhance their opportunities to define smart and reliable e-health systems. This comes across with the need for developing a comprehensive and suitable solution to handle security issues within IoMT platforms. We presented in this manuscript the IoMT-CySAM, our solution for effectively assessing and managing cyber risks in IoMT systems. By integrating various techniques, it allows to easily put into practice our approach for evaluating trustworthiness and managing cyber security risks within IoMT environments.

As primary future work, we plan to focus on deploying the system in real-world environments. This will enable us to evaluate its performance across diverse operational scenarios, analyze its scalability and limitations during real-time execution, and assess how effectively it integrates with existing solutions.

## References

[1] Guntur SR, Gorrepati RR, Dirisala VR (2019). Robotics in healthcare: an internet of medical robotic things (IoMRT) perspective. Machine Learning in Bio-Signal Analysis and Diagnostic Imaging.

[2] Ksibi S, Jaidi F, Bouhoula A (2022). A comprehensive study of security and cyber-security risk management within e-health systems: synthesis, analysis and a novel quantified approach. Mob Netw Appl.

[3] Yadav S (2024). Transformative frontiers: a comprehensive review of emerging technologies in modern healthcare. Cureus.

[4] Ksibi S, Jaidi F, Bouhoula A (2024). oMT applications perspectives: from opportunities and security challenges to cyber-risk management. Decision Making and Security Risk Management for IoT Environments. Advances in Information Security.

[5] Houichi M, Jaidi F, Bouhoula A (2024). Cyber security within smart cities: a comprehensive study and a novel intrusion detection-based approach. Comput Mater Contin.

[6] Srivastava P, Khan R (2018). A review paper on cloud computing. Int J Adv Sci Comp Eng.

[7] Hameed K, Naha R, Hameed F (2024). Digital transformation for sustainable health and well-being: a review and future research directions. Discov Sustain.

[8] Selvaraj S, Sundaravaradhan S (2020). Challenges and opportunities in IoT healthcare systems: a systematic review. SN Appl Sci.

[9] Bhuiyan MN, Rahman MM, Billah MM (2021). Internet of things (IoT): a review of its enabling technologies in healthcare applications, standards protocols, security, and market opportunities. IEEE Internet Things J.

[10] Bhatt MW, Sharma S (2023). An IoMT-based approach for real-time monitoring using wearable neuro-sensors. J Healthc Eng.

[11] AlShorman O, AlShorman B, Alkhassaweneh M, Alkahtani F (2020). A review of internet of medical things (IoMT)-based remote health monitoring through wearable sensors: a case study for diabetic patients. Indones J Electr Eng Comput Sci.

[12] Kashani MH, Madanipour M, Nikravan M (2021). A systematic review of IoT in healthcare: applications, techniques, and trends. J Netw Comput Appl.

[13] Van Gemert-Pijnen J, Peters O, Ossebaard HC (2013). Improving Ehealth. https://books.google.tn/books?id=mU1-mwEACAAJ.

[14] (2024). Internet of Things [IoT] Market Size, Share and Growth by 2030. https://www.fortunebusinessinsights.com/industry-reports/internet-of-things-iot-market-100307.

[15] BS ISO 31000:2009 (2025). ISO 31000 Risk Management - Principles and Guidelines. https://pecb.com/whitepaper/iso-31000-risk-management--principles-and-guidelines.

[16] Joint Task Force (2018). Risk Management Framework for Information Systems and Organizations: A System Life Cycle Approach for Security and Privacy. NIST Special Publication 800-37
Revision 2. NIST Special Publication.

[17] International Organization for Standardization (2025). ISO 14971:2019 - Medical devices: Application of risk management to medical devices. https://www.iso.org/standard/72704.html.

[18] International Organization for Standardization (2025). ISO/TR 24971:2020. Medical devices — Guidance on the application of ISO 14971. https://www.iso.org/standard/74437.html.

[19] International Electrotechnical Commission (2025). IEC 80001-1:2021. Application of risk management for IT-networks incorporating medical devices. Part 1: Safety, effectiveness and security in the implementation and use of connected medical devices or connected health software. https://www.iso.org/standard/72026.html.

[20] Joint Task Force (2012). NIST SP 800-30 Rev. 1 - Guide for Conducting Risk Assessments.

[21] Souppaya M, Scarfone K (2016). NIST SP 800-154 - Guide to Data-Centric System Threat Modeling. https://csrc.nist.gov/files/pubs/sp/800/154/ipd/docs/sp800_154_draft.pdf.

[22] Boeckl K, Boeckl K, Fagan M (2019). NISTIR 8228 - Considerations for managing Internet of Things (IoT) cybersecurity and privacy risks.

[23] OWASP OWASP, CSA CSA (2025). OWASP Secure Medical Devices Deployment Standard. https://cloudsecurityalliance.org/artifacts/owasp-secure-medical-devices-deployment-standard.

[24] Freund J, Jones J (2014). Measuring and Managing Information Risk: A FAIR Approach. https://dl.acm.org/doi/10.5555/2829021.

[25] (2024). MITRE Corporation. MITRE ATT&CK Framework. https://attack.mitre.org.

[26] Ksibi S, Jaidi F, Bouhoula A (2021). Cyber-Risk Management within IoMT: A Context-Aware Agent-Based Framework for a Reliable e-Health System. Proceedings of the 23rd International Conference on Information Integration and Web Intelligence.

[27] Ksibi S, Jaidi F, Bouhoula A (2023). IoMT Security Model based on Machine Learning and Risk Assessment Techniques. Proceedings of the 2023 IEEE International Wireless Communications and Mobile Computing Conference (IWCMC).

[28] Freyer O, Jahed F, Ostermann M, Rosenzweig C, Werner P, Gilbert S (2024). Consideration of cybersecurity risks in the benefit-risk analysis of medical devices: scoping review. J Med Internet Res.

[29] Messinis S, Temenos N, Protonotarios NE, Rallis I, Kalogeras D, Doulamis N (2024). Enhancing Internet of Medical Things security with artificial intelligence: a comprehensive review. Comput Biol Med.

[30] Ksibi S, Jaidi F, Bouhoula A (2025). MLRA-Sec: an adaptive and intelligent cyber-security-assessment model for internet of medical things (IoMT). Int J Inf Secu.

